# Mood alterations in mouse models of Spinocerebellar Ataxia type 1

**DOI:** 10.1038/s41598-020-80664-9

**Published:** 2021-01-12

**Authors:** Melissa Asher, Juao-Guilherme Rosa, Marija Cvetanovic

**Affiliations:** 1 Department of Neuroscience, University of Minnesota, 2101 6th Street SE, Minneapolis, MN 55455 USA; 2 Institute for Translational Neuroscience, University of Minnesota, 2101 6th Street SE, Minneapolis, MN 55455 USA

**Keywords:** Diseases of the nervous system, Emotion

## Abstract

Spinocerebellar ataxia type 1 (SCA1) is a fatal neurodegenerative disease caused by abnormal expansion of glutamine-encoding CAG repeats in the *Ataxin-1 (ATXN1*) gene. SCA1 is characterized by progressive motor deficits, cognitive decline, and mood changes including anxiety and depression, with longer number of repeats correlating with worse disease outcomes. While mouse models have been very useful in understanding etiology of ataxia and cognitive decline, our understanding of mood symptoms in SCA1 has lagged. It remains unclear whether anxiety or depression stem from an underlying brain pathology or as a consequence of living with an untreatable and lethal disease. To increase our understanding of the etiology of SCA1 mood alterations, we used the elevated-plus maze, sucrose preference and forced swim tests to assess mood in four different mouse lines. We found that SCA1 knock-in mice exhibit increased anxiety that correlated with the length of CAG repeats, supporting the idea that underlying brain pathology contributes to SCA1-like anxiety. Additionally, our results support the concept that increased anxiety is caused by non-cerebellar pathology, as Purkinje cell specific SCA1 transgenic mice exhibit *decreased* anxiety-like behavior. Regarding the molecular mechanism, partial loss of ATXN1 may play a role in anxiety, based on our results for Atxn1 haploinsufficient and null mice.

## Introduction

Spinocerebellar ataxia type 1 (SCA1) belongs to the group of the spinocerebellar ataxias (SCAs), autosomal dominantly inherited cerebellar neurodegenerative diseases with progressive ataxia. SCA1 is caused by an abnormal expansion of CAG repeats in the coding region of *Ataxin-1* (*ATXN1)* gene, which leads to an expanded polyglutamine (polyQ) tract in the protein ATXN1^1^. While the non-pathogenic *ATXN1* allele contains 6–38 CAG repeats interrupted by a CAT sequence, mutation carriers have 39–82 continuous CAG repeats. These repeats are unstable and can undergo further germline expansion, often resulting in larger number of repeats in the offspring that correlates with earlier onset and more severe disease in successive generations^2,3^.

SCA1 is primarily diagnosed by the onset of progressive movement deficits (ataxia) in the patient’s mid-thirties on average^4^. In addition, many patients also experience cognitive symptoms^5–7^ and mood alterations, including anxiety, depression, and suicidal thoughts^8^.

Based on a large EUROSCA study that included patients with SCA 1, 2, 3, and 6, approximately half of SCA patients reported depression or anxiety using the quality of life (EQ-5D mood) questionnaire, and 17% and 15% of patients reported depression and anxiety in the patient health questionnaire (PHQ) (21.9 and 15% in SCA1)^9^. While follow-up studies using multivariate analysis found associations between ataxia and depression in SCA^8,10^, other studies using different measures of depression (Beck Depression Inventory) found that motor and dominant hand functions were more predictive of depressed mood than overall ataxia score^6^. A longitudinal EUROSCA Natural History study linked faster decline in mood alterations in patients with cognitive impairments^11^. While the reported percentage of patients exhibiting depression, anxiety, and suicidal thoughts varied between studies (from 15 to 50%), these alterations in affect were found to significantly impair the quality of life and disease outcomes of SCA patients even after accounting for the severity of ataxia^9,12–15^.

We currently have a good understanding of the pathological mechanisms of ataxia in SCA1^3^. It is generally accepted that cerebellar pathology caused by a toxic gain of mutant ATXN1 function is a major contributor to motor deficits in SCA1^16^. Recent studies also indicate that the cerebellum contributes to cognitive deficits in SCA1^17^, while brain stem and spinal cord pathologies contribute to premature lethality^18^. In contrast, very little is known about the etiology of mood symptoms in SCA1. For example, it is not clear whether anxiety and depression stem from the knowledge that patients suffer from untreatable, progressively debilitating and fatal disease, or is caused by underlying pathology^6,8,19^. Moreover, it is unclear whether anxiety and depression in patients with SCAs are caused by cerebellar neurodegeneration or pathology in other brain regions^20^. In addition to its well-known role in motor movement, cerebellar contribution to cognition and mood has been increasingly recognized^21,22^. Injury limited to the cerebellum can cause similar spectrum of cognitive and mood alterations, including increased anxiety, and depression, termed Cerebellar Cognitive Affective Syndrome (CCAS)^23,24^. As depression and anxiety impact the patient’s and their caregiver’s quality of life, it is important to understand their causes so that comprehensive therapies can be developed to treat all aspects of SCA1^12^.

Mouse models of SCA1 have been essential in implicating cerebellar pathology and gain-of-function mechanisms in ataxia and cognitive deficits^17,25–27^. For instance, motor deficits in the transgenic SCA1 mouse line *ATXN1[82Q]*, which overexpress mutant ATXN1[82Q] protein selectively in cerebellar Purkinje cells, provided support for the predominant role of cerebellar dysfunction in ataxia^28^. In addition, motor symptoms in two SCA1 knock-in mouse lines (*Atxn1*^*154Q/2Q*^ and *Atxn1*^*78Q/2Q*^) correlate with the number of polyQ repeats, similar to symptoms in patients with SCA1^26,29^. Regarding the underlying molecular mechanism, the concept that polyQ expansion causes ataxia largely through a toxic gain-of-function mechanism is supported by the lack of ataxia in Atxn1 null mice^27,30,31^.

However, to date there has been no systematic investigation of mood in these mouse models. We wished to determine (1) whether SCA1 mice exhibit mood alterations, (2) whether these alterations correlate with the number of polyQ repeats, and whether (3) cerebellar pathology and (4) loss of ATXN1 function contribute to mood alterations. This report represents our observations in four mouse lines whose performance on elevated plus maze, forced swim test and sucrose preference was examined to address these questions.

## Results

### Ataxin-1 knock-in mice exhibit increased anxiety and sucrose consumption indicating that mutant Ataxin-1 induced pathogenesis may cause mood alterations

We first tested mood in *Atxn1*^*154Q/2Q*^ knock-in mice, in which one *Atxn1* allele has a long CAG expansion (154 repeats)^26^, while the other allele has 2 CAG repeats which is normal for wild-type mice. In these mice the mutant gene is under the control of its endogenous promoter maintaining the brain wide expression of mutant ATXN1 at physiological levels. Importantly, we started testing *Atxn1*^*154Q/2Q*^ mice at 8 weeks of age, prior to detectable ataxia (at 12 weeks) or failure to gain weight (11 weeks) to minimize potential interference of motor deficits or alterations in weight with mood testing^32^.

**Figure 1:**
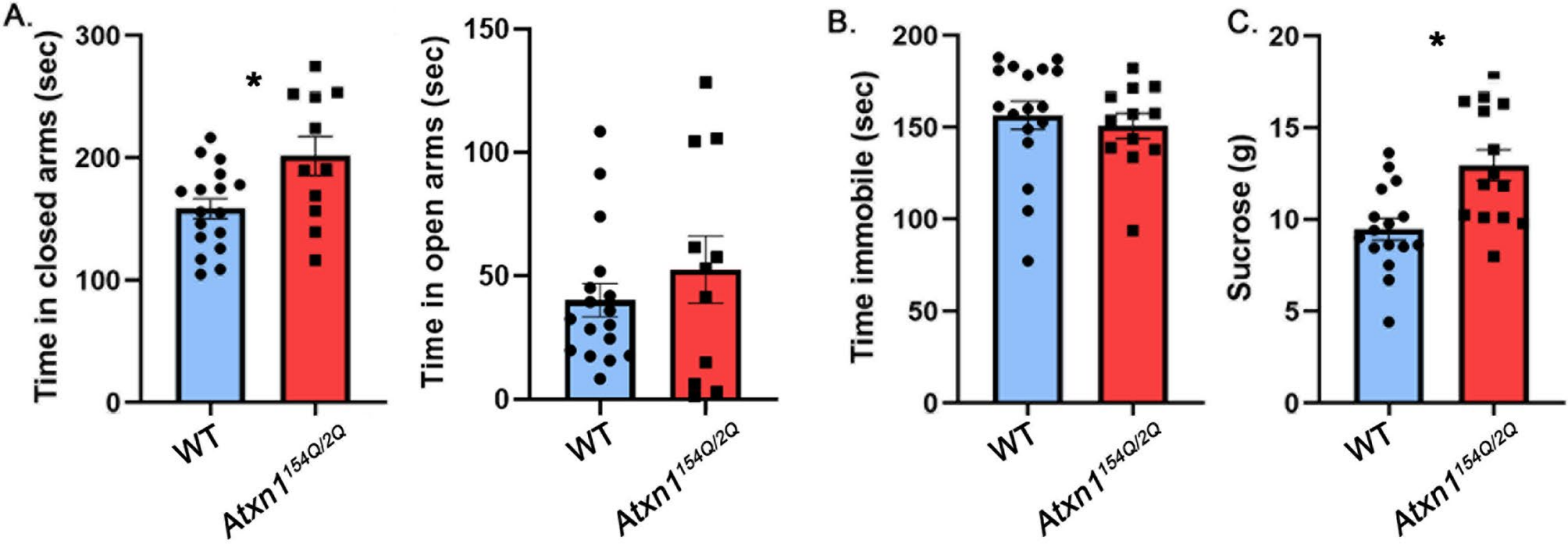
*Atxn1*^*154Q/2Q*^ mice exhibit anxiety-like phenotype. 8–10 weeks old *Atxn1*^*154Q/2Q*^ mice and their wild-type littermates were tested on the Elevated Plus Maze, Forced Swim Test and Sucrose preference test. (**A**) Time in closed and open arms. (**B**) Time spent immobile. (**C**) Amount of sucrose consumed. * indicates *P* < 0.05 using unpaired, two-tailed t test, data are presented as mean ± SEM.

The elevated plus maze (EPM) is a test used to assess anxiety-like responses of rodents^33^. The EPM relies upon rodents’ proclivity toward dark, enclosed spaces and an unconditioned fear of heights/open spaces. It consists of an elevated maze with four arms (two open and two enclosed) that are arranged to form a plus shape, and anxiety behavior is assessed as increased time spent in the closed arms^33^. *Atxn1*^*154Q/2Q*^ mice spent more time in the closed arms than their wild-type littermate controls (Fig. 1A left, 27.2% increase with 95% confidence interval of (6.3%, 48.3%), wild-type (WT) average 158.3 ± 8.19 s, *Atxn1*^*154Q/2Q*^ average 201.4 ± 15.96 s, *P* = 0.0138, unpaired, two-tailed t-test, N = 17 WT and 11 *Atxn1*^*154Q/2Q*^ mice), suggesting an anxiety-like phenotype. They also spent less time in the open arms than their wild-type littermate controls but this difference was not statistically significant (wild-type (WT) average 52.53 ± 13.52 s, *Atxn1*^*154Q/2Q*^ average 40.26 ± 6.64 s, *P* = 0.3757, unpaired, two-tailed t-test, N = 17 WT and 11 *Atxn1*^*154Q/2Q*^ mice).

The forced swim test (FST) is commonly used for depression-like behavior. The FST involves the scoring of active (swimming and climbing) versus passive (immobility) behavior when mice are forced to swim in a cylinder from which there is no escape^34^. *Atxn1*^*154Q/2Q*^ mice were indistinguishable from wild-type littermate controls in immobility time (Fig. 1B, − 3.7% effect size with 95% confidence interval (− 17.8%, 10.4%), WT average 156.6 ± 7.64 s, *Atxn1*^*154Q/2Q*^ median 150.8 ± 6.7 s, *P* = 0.595 unpaired, two-tailed t-test, N = 17 WT and 12 *Atxn1*^*154Q/2Q*^ mice).

Anhedonia is the inability to experience pleasure from rewarding or enjoyable activities and is a core symptom of depression in humans. To measure anhedonia in mice, we used a sucrose preference test (SPT) based on a two-bottle choice paradigm in which mice were offered water in one bottle and sucrose in the other. SPT relies on the ability of sucrose to act as a natural reward, and a reduction in sucrose preference indicates anhedonia and depression^35^. However it is important to note that additional parameters, including alterations in metabolism^36^, changes in weight or disruption of reward system^37^ may confound this interpretation. Contrary to our expectations, we found that *Atxn1*^*154Q/2Q*^ mice consumed significantly *more* sucrose than wild-type littermates (Fig. 1C, 36.8% increase with 95% confidence interval (14.9%, 58.6%), WT average 9.46 ± 0.58 g, *Atxn1*^*154Q/2Q*^ average 12.95 ± 0.84 g, N = 16 WT and 12 *Atxn1*^*154Q/2Q*^ mice *P* = 0.0018, unpaired, two-tailed t-test), while water consumption stayed the same (WT average 4.009 g, *Atxn1*^*154Q/2Q*^ average 3.199 g, *P* = 0.6786). While this may be interpreted as a lack of depression-like phenotype, alternatively mice may already exhibit alterations in metabolism^38^ or expression of genes or circuits important for the reward system^39,40^ that affected their sucrose consumption. It is also important to note that using anhedonia may not be the best way to evaluate depression in SCA1 as there is no difference in depression prevalence between patients with SCA1 that have decreasing or increasing body mass index (50% and 51.4% respectively)^15^.

There were no differences between female and male mice in any of the tests.

These results indicate that *Atxn1*^*154Q/2Q*^ mice exhibit increased anxiety-like behavior but not a typical depressive-like phenotype, as they had increased sucrose preference. They support the concept that anxiety in patients with SCA1 may be caused, at least in part, by underlying brain pathology.

### Anxiety-like behavior correlates with number of polyQ repeats

The onset and severity of motor deficits in patients with SCA1 correlate with the number of polyQ repeats in ATXN1, with higher numbers of repeats causing earlier onset and more severe symptoms^2^. Two knock-in mouse models of SCA1 created to express ATXN1 with 154Q and 78Q, *Atxn1*^*154Q/2Q*^ and *Atxn1*^*78Q/2Q*^ respectively, exhibit a similar correlation between the length of polyQ repeats and severity of motor deficits, albeit at longer polyQ repeats than those found in humans. For example, onset of ataxia, as measured by decreased rotarod performance, occurs at 12 weeks in *Atxn1*^*154Q/2Q*^ mice, while *Atxn1*^*78Q/2Q*^ mice show milder rotarod deficits only at 6 months^29^. Therefore, we next tested whether mood alterations in SCA1 knock-in mice similarly depend on the length of the polyQ expansion by evaluating performance of *Atxn1*^*78Q/2Q*^ mice on mood tests.

**Figure 2:**
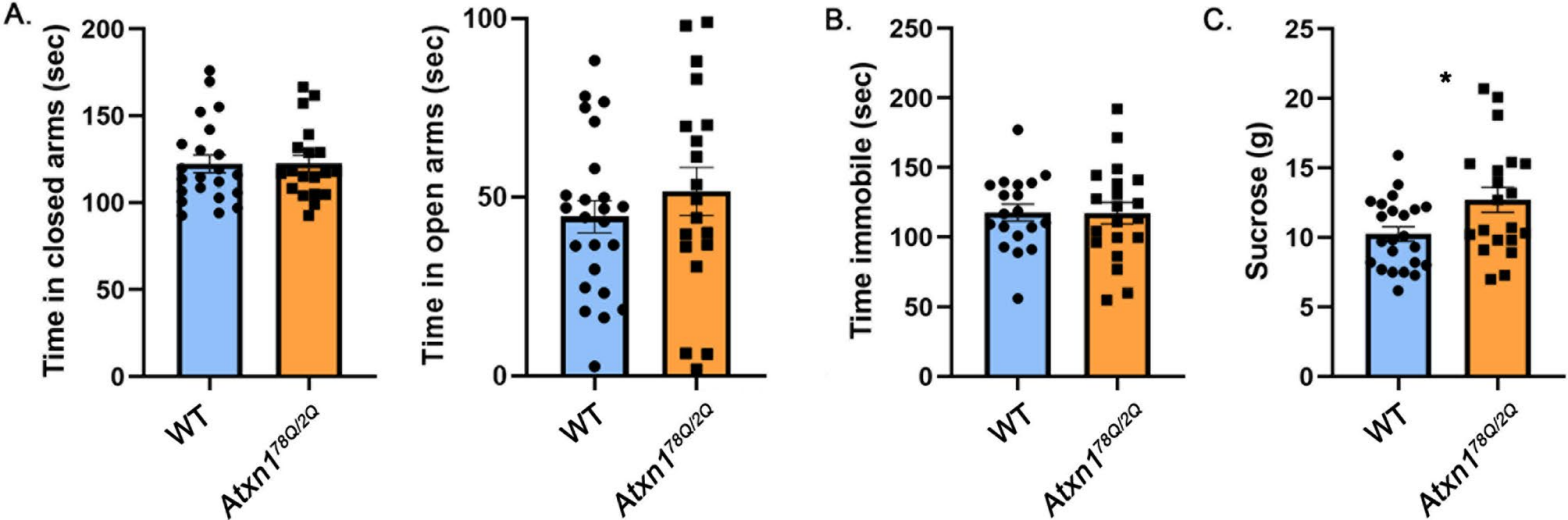
*Atxn1*^*78/2Q*^ mice exhibit increased sucrose consumption. 8–10 weeks old *Atxn1*^*78Q/2Q*^ mice and their wild-type littermates were tested on the Elevated Plus Maze, Forced Swim Test and Sucrose preference test. (**A**) Time in closed and open arms. (**B**) Time spent immobile. (**C**) Amount of sucrose consumed. * indicates *P* < 0.05 using unpaired, two-tailed t test, data are presented as mean ± SEM.

There was no significant difference between *Atxn1*^*78Q/2Q*^ and wild-type mice on the elevated plus maze (Fig. 2A, 0.2% effect size with 95% confidence interval (− 11.5%, 11.8%), WT average time in closed arms = 122.3 ± 5.079 s, *Atxn1*^*78Q/2Q*^ average time in closed arms = 122.5 ± 4.718 s, N = 22 WT and 19 *Atxn1*^*78Q/2Q*^ mice, *P* = 0.9724, unpaired, two-tailed t-test, WT average time in open arms = 51.57 ± 6.73 s, *Atxn1*^*78Q/2Q*^ = 44.56 ± 4.5 s, *P* = 0.3765, unpaired, two-tailed t-test), suggesting that knock-in SCA1 mice expressing mutant ATXN1 with fewer polyQ repeats do not exhibit an anxiety-like phenotype at this age.

*Atxn1*^*78Q/2Q*^ mice were also indistinguishable from WT mice on the forced swim test (Fig. 2B, − 0.4% effect size with 95% confidence interval (− 17.4%, 16.7%), WT average time immobile = 117.6 ± 6.13 s, *Atxn1*^*78Q/2Q*^ average time immobile = 117.2 ± 7.73 s, N = 19 WT and 20 *Atxn1*^*78Q/2Q*^ mice, Student’s unpaired two-tailed t-test *P* = 0.9661).

However, *Atxn1*^*78Q/2Q*^ mice consumed *more* sucrose than wild-type littermate controls (Fig. 2C, 24.4% effect size with 95% confidence interval (4%, 44.8%), WT average 10.23 ± 0.52 g, *Atxn1*^*78Q/2Q*^ average 12.7 ± 0.38 g, N = 23 WT and 20 *Atxn1*^*78Q/2Q*^ mice, *P* = 0.0194, unpaired, two-tailed t-test) with no statistically significant difference in water consumption (WT average 5.21 g, *Atxn1*^*78Q/2Q*^ average 6.13 g, *P* = 0.5498). This suggests that increased sucrose consumption, unlike other features of the *Atxn1*^*154Q/2Q*^ model, can be caused by the shorter polyglutamine tract.

### Purkinje neuron specific transgenic SCA1 mice exhibit reduced anxiety

One of the hallmarks of SCA1 is pronounced cerebellar pathology, with significant loss of Purkinje neurons in the cerebellar cortex^41^. Increasing evidence supports the role of the cerebellum in mood in addition to its role in motor control^42^. For example, patients with the cerebellar injury exhibit disinhibition and other alterations in mood, collectively termed Cerebellar Cognitive Affective Syndrome (CCAS)^23,43^.

To investigate the contribution of cerebellar pathology to the mood alterations caused by mutant ATXN1, we examined an SCA1 transgenic mouse model, *ATXN1[82Q]* mice, which overexpress mutant ATXN1[82Q] only in cerebellar Purkinje cells under the control of the Purkinje cell protein 2 (Pcp2) promoter^28^.

**Figure 3:**
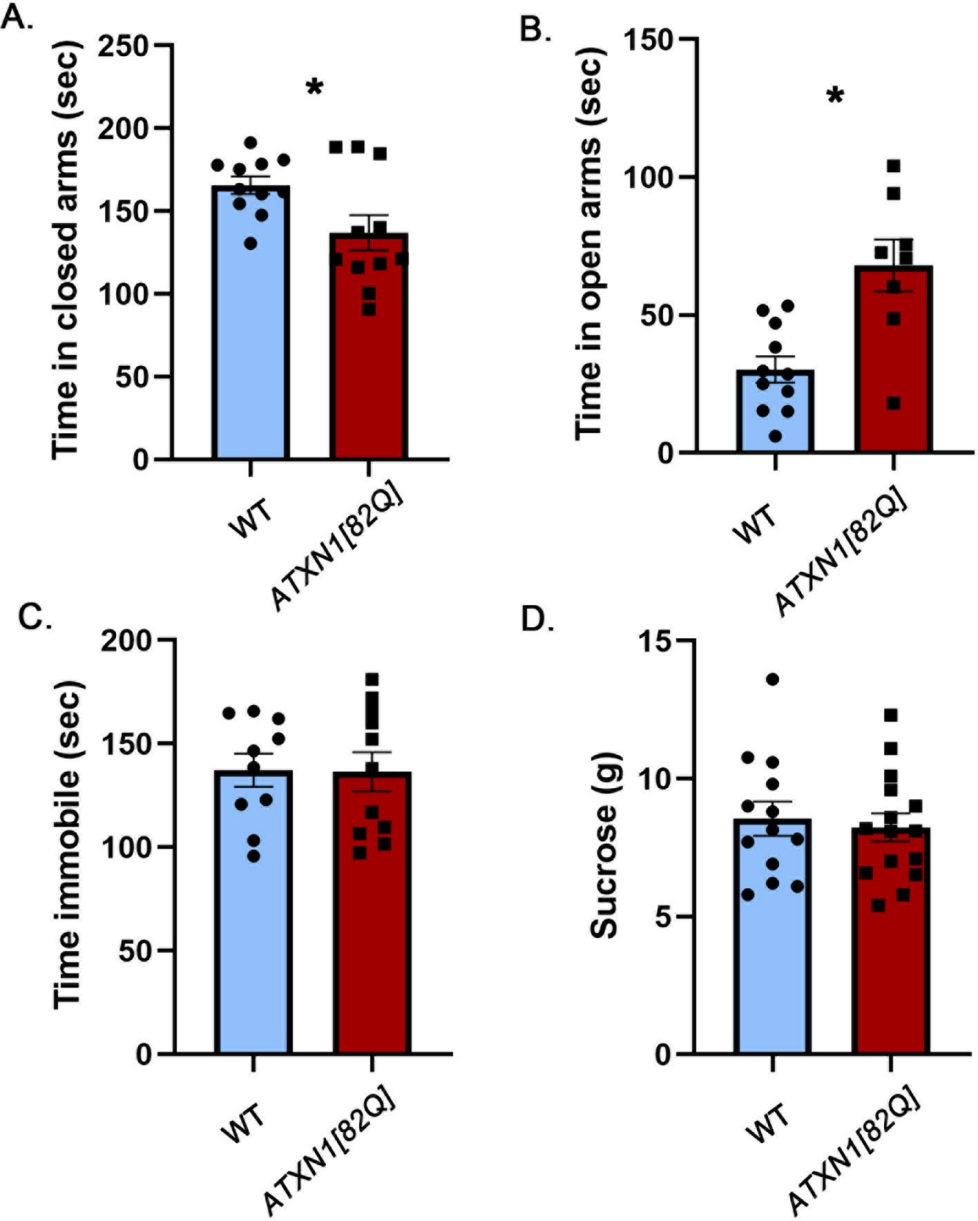
Cerebellar Purkinje cell-specific *ATXN1[82Q]* transgenic mice have attenuated anxiety. 8–10 weeks old *ATXN1[82]* mice and their wild-type littermates were tested on the Elevated Plus Maze, Forced Swim Test and Sucrose preference test. (**A**) Time in closed arms. (**B**) Time in open arms. (**C**) Time spent immobile. (**D**) Amount of sucrose consumed. * indicates *P* < 0.05 using the unpaired, two-tailed t test, data are presented as mean ± SEM.

Surprisingly, we found a striking *reduction* in anxiety-like behavior in the *ATXN1[82Q]* mice on the elevated plus maze: they spent significantly less time in the closed arms (Fig. 3A, − 17.3% effect size with 95% confidence interval (− 32.5%, − 2.3%), WT average 165.6 ± 5.257 s, *ATXN1[82Q]* average 137 ± 10.58 s, N = 11 WT and 8 *ATXN1[82Q]* mice, *P* = 0.0253, unpaired, two-tailed t-test). This was accompanied by increased time in the open arms, another sign of decreased anxiety (Fig. 3B, 125% effect size with 95% confidence interval (56.9%, 193%), WT average 30.22 ± 4.745 s, *ATXN1[82Q]* average 67.99 ± 9.448 s, *P* = 0.0012, unpaired, two-tailed t-test).

We did not observe any differences between *ATXN1[82Q]* and wild-type mice in either the forced swim test (Fig. 3C, − 0.5% effect size with 95% confidence interval (− 19.6%, 18.5%), WT average 137.3 ± 8.064 s, *ATXN1[82Q]* average 136.5 ± 9.38 s, N = 11 WT and 10 *ATXN1[82Q]* mice, *P* = 0.9516, unpaired, two-tailed t-test) or the sucrose preference test (Fig. 3D, − 3.75% effect size with 95% confidence interval (− 22.7%, 15.2%), WT average 8.55 ± 0.6187 g, *ATXN1[82Q]* average 8.523 ± 0.5037 g, N = 13 WT and 15 *ATXN1[82Q]* mice, *P* = 0.6882, unpaired, two-tailed t-test).

**Figure 4:**
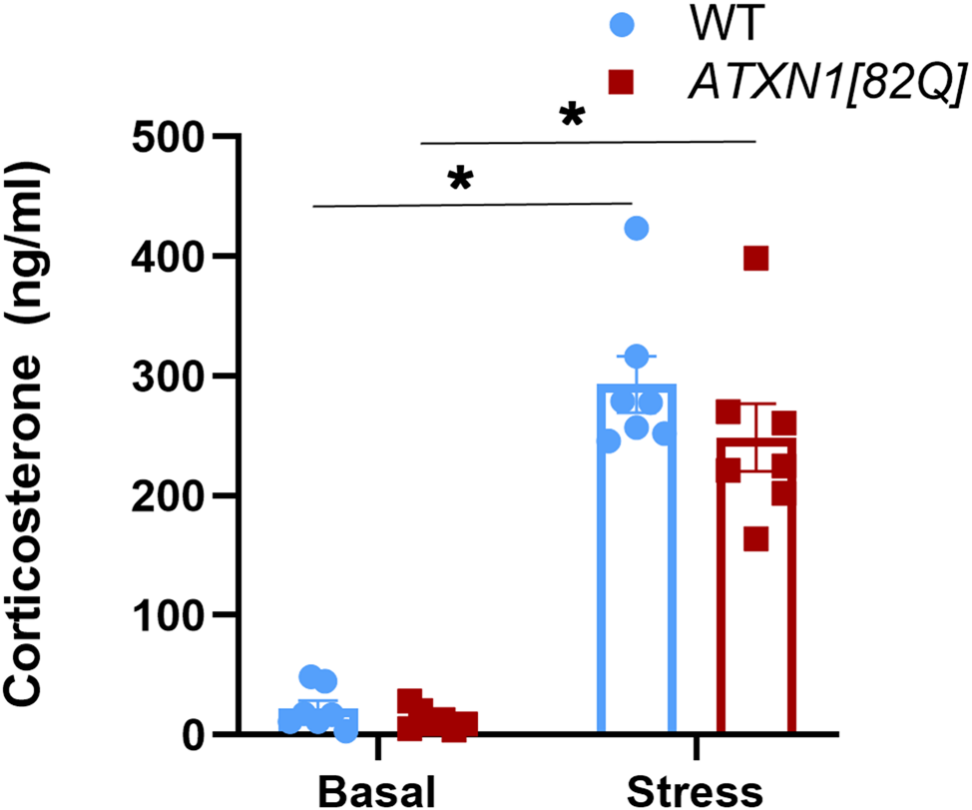
Basal and stress-induced blood corticosterone levels in *ATXN1[82Q]* and wild-type control mice. Corticosterone levels were determined in total plasma from 7 pairs of *ATXN1[82Q]* mice and littermate WT controls. Basal/minimally stressed plasma was obtained in the morning (9–12 am) at least three days before stress induction. Stress level blood samples were collected after 15 min of restraint stress. * indicates *P* < 0.05 using the unpaired, two-tailed t test, data are presented as mean ± SEM.

To test whether attenuated anxiety *ATXN1[82Q]* mice is caused by a reduced stress-induction, we examined both basal and restraint stress-induced blood corticosterone levels in *ATXN1[82Q]* mice and wild-type littermate controls. We found a trend towards decreased basal and stress-induced corticosterone levels, although this difference was not statistically significant (Fig. 4, basal WT average 21.81 ± 6.64, basal *ATXN1[82Q]* average 12.55 ± 3.27, stress induced WT average 293 ± 23.54, stress-induced *ATXN1[82Q]* average 248 ± 28.35, two way ANOVA significant effect of stress *P* < 0.0001 but not of genotype).

These results indicate that cerebellar dysfunction may attenuate anxiety in mice, akin to disinhibition seen in patients with cerebellar injury. Moreover, these results are in contrast to the increased anxiety seen in SCA1 knock-in mice with brain-wide expression of mutant polyQ ATXN1, suggesting that non-cerebellar pathology plays a dominant role in SCA1-like anxiety.

### Partial and full loss of ataxin-1 cause mood alterations in mice

PolyQ expansion in ATXN1 leads to both gain and loss of ATXN1 function^44,45^. Gain-of-function is the predominant molecular mechanism of motor deficits in SCA1, as supported by the lack of a strong motor phenotype in Atxn1 null mice^25,27^, while loss-of-function may contribute to cognitive deficits^17,27^. We therefore wanted to determine whether loss of ataxin-1 alters mood in *Atxn1*^*−/−*^ mice. Additionally, patients with SCA1 have one normal and one mutant allele, and a simple loss-of-function molecular mechanism would result in an approximately 50% decrease in ATXN1 function. To examine the effects of an approximately 50% loss of ataxin-1 function we also analyzed mood in heterozygous knockout *(Atxn1*^+*/−*^*)* mice.

**Figure 5:**
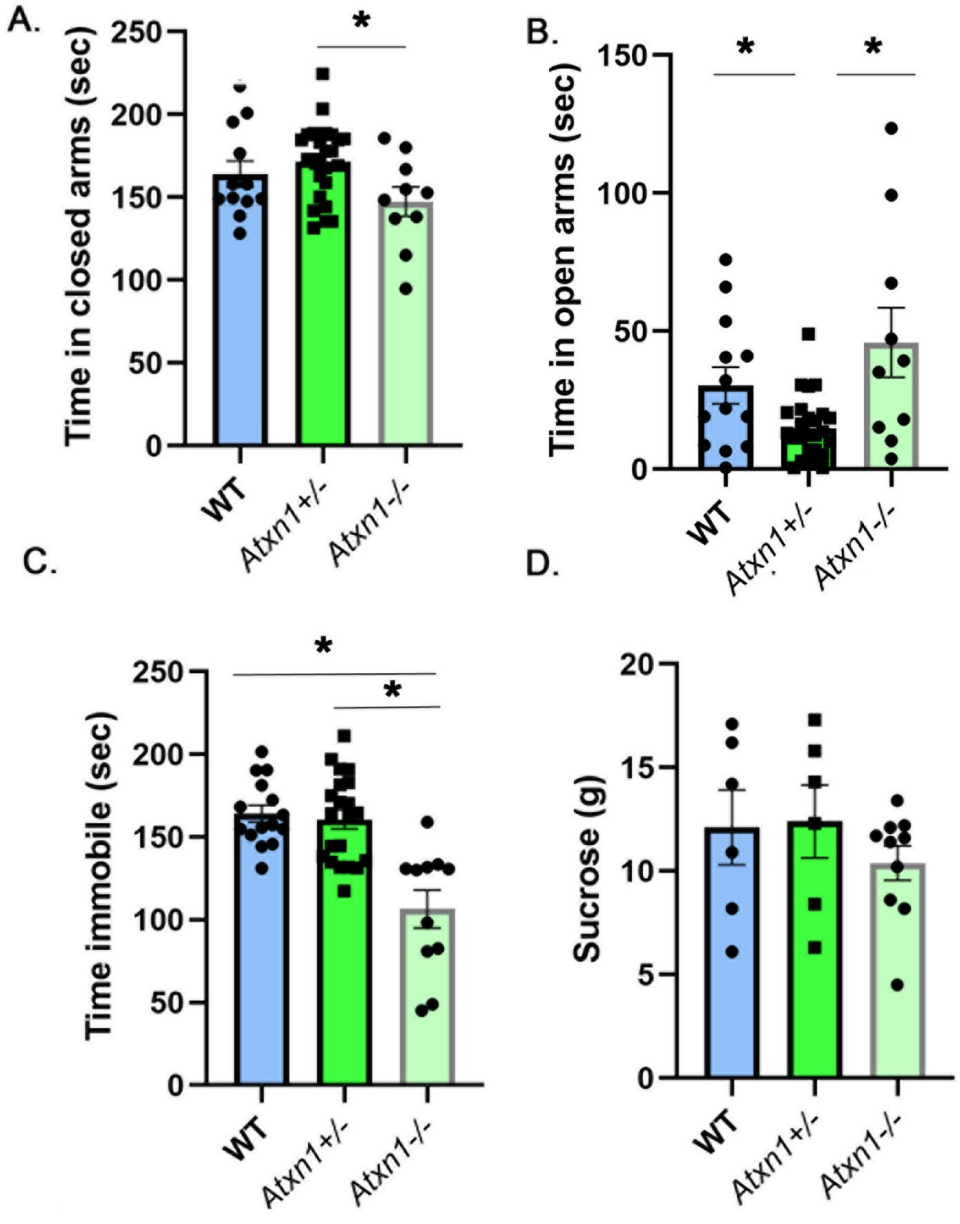
Decreased immobility in homozygous (*Atxn1*^*−/*−^) and increased anxiety in heterozygous (*Atxn1*^+*/-*^) mice. 8–10 weeks old wild-type, *Atxn1*^+*/−*^*,* and *Atxn1*^*−/*−^ mice were tested on the Elevated Plus Maze, Forced Swim Test and Sucrose preference test. (**A**) Time in closed arms. (**B**) Time in open arms. (**C**) Time spent immobile. (**D**) Amount of sucrose consumed. * indicates *P* < 0.05 using the One way ANOVA followed by post-hoc Tukey’s testing, data are presented as mean ± SEM.

On the elevated plus maze, heterozygous *Atxn1*^+*/−*^ mice spent slightly more time in the closed arms than wild-type or null mice (Fig. 5A, 9.45% and 16.4% effect sizes with 95% confidence intervals (− 3.2%, 21.3%) and (2.3%, 31.3%) respectively comparing *Atxn1*^+/−^ to WT and *Atxn1*^*-/−*^ mice, WT mice average 156.3 ± 9.19 s, *Atxn1*^+/−^ average 171.6 ± 4.97 s, *Atxn1*^*−/−*^ average 147.4 ± 5.72 s, N = 13 WT, 26 *Atxn1*^+*/−*^, and 10 *Atxn1*^*−/−*^ mice, One-way ANOVA, F_(2,44)_ = 3.34, *P* = 0.044) but this difference was statistically significant only when compared to null mice (*P* = 0.14 and *P* = 0.034 One-way ANOVA followed by post-hoc Tukey’s multiple comparison test comparing *Atxn1*^+/−^ to WT and *Atxn1*^*−/−*^ mice respectively). Additionally, *Atxn1*^+*/−*^ mice spent less time in the open arms than wild-type or null mice (Fig. 5B, − 104.4% and − 210% effect sizes with 95% confidence intervals (− 180.80%, − 29.3%) and (− 349%, − 70%) respectively comparing *Atxn1*^+/−^ to WT and *Atxn1*^*−/−*^ mice, WT mice average 30.2 ± 6 s, *Atxn1*^+/−^ average 14.81 ± 2.21 s, *Atxn1*^*−/−*^ average 45.92 ± 12 s, F_(2,44)_ = 7.04, *P* = 0.0021, One-way ANOVA followed by post-hoc Tukey’s multiple comparison tests *P* = 0.01 and 0.022 comparing *Atxn1*^+/−^ to WT and *Atxn1*^*−/−*^ mice). These results indicate an increased anxiety-like phenotype with 50% loss of ATXN1.

Full loss of ATXN1 resulted in slightly decreased anxiety in Atxn1 null mice compared to wild-type littermates, i.e. increased time in open arms (effects size of 34.8% with 95% confidence interval (− 13.4%, 81.6%), WT mice average 30.2 ± 6 s, *Atxn1*^*−/−*^ average 45.92 ± 12 s) and decreased time in closed arms (− 10.2% effect size with 95% confidence interval (− 5.6%, 25.9%), WT mice average 156.3 ± 9.19 s, *Atxn1*^*−/−*^ average 147.4 ± 5.72 s), but this was not statistically significant (one-way ANOVA with post-hoc Tukey’s test, *P* = 0.2745, and *P* = 0.2476 respectively).

This decrease in inhibition is consistent with the forced swim test, where Atxn1 null mice were significantly less immobile (Fig. 5C, − 54.1% and − 50.8% effect sizes with 95% confidence intervals (− 77.4%, − 30.1%) and (− 72.7%, − 28.9%) respectively comparing *Atxn1*^−/−^ to WT and *Atxn1*^+*/−*^ mice, WT average 164.2 ± 5.093 s, *Atxn1*^+*/−*^ average 160.6 ± 5.73 s, *Atxn1*^*−/−*^ average 106.5 ± 11.39 s, N = 15 WT, 21 *Atxn1*^+*/−*^, and 11 *Atxn1*^*−/−*^ mice, one-way ANOVA F_(2,44)_ = 17.17, followed by post-hoc Tukey’s multiple comparison tests *P* < 0.0001 for *Atxn1*^*−/*−^ vs. both WT and *Atxn1*^+*/−*^).

While *Atxn1*^*−/−*^ mice consumed slightly less sucrose on average (Fig. 5D, − 16.5% and − 19.36% effect sizes with 95% confidence intervals (− 60.3%, 26.9%) and (− 62.9%, 24.1%), WT mice 12.12 ± 1.81 g, *Atxn1*^+*/−*^ 12.4 ± 1.75 g, *Atxn1*^*−/−*^ 10.39 ± 0.83 g) these differences were not significant (one-way ANOVAF_(2,19)_ = 0.687, *P* = 0.51, followed by post-hoc Tukey’s multiple comparison tests *P* = 0.6369 and *P* = 0.5461 respectively for Atxn1^−/−^ vs. WT and *Atxn1*^+*/−*^).

Together these results indicate that full and partial loss of ATXN1 alter mood phenotype in mice in opposite ways, with partial loss leading to increased anxiety and full loss resulting in decreased anxiety.

## Discussion

In order to gain insight into the etiology of SCA1 mood alterations, we systematically (e.g. using the same tests on the same genetic background at 8 weeks of age) investigated how mood in mice was affected by global or cerebellum-limited expression of mutant ATXN1, polyQ length of ATXN1, and loss of ATXN1. In order to minimize the confounding effect of impaired movement on behavioral tests, we have chosen 8 weeks as the testing age because it precedes motor deficits. In addition, there is no detectable neurodegeneration at this age^27,29,32,46–48^ (e.g. reproducible dendritic atrophy and neuronal loss in the cerebellum are detected at 12 weeks and 24 weeks respectively in *Atxn1*^*154Q/2Q*^ and *ATXN1[82Q]* mice, whereas neuronal loss in the hippocampus is seen at 5 months in *Atxn1*^*154Q/2Q*^ mice. No neurodegeneration has been described in *Atxn1−/− *and *Atxn1*^*78Q/2Q*^ lines).

We found that *Atxn1*^*154Q/2Q*^ mice, an SCA1 knock-in line with global expression of mutant ATXN1, exhibit an anxiety-like phenotype on the elevated plus maze. This suggests that anxiety seen in patients with SCA1 may be, at least in part, caused by underlying pathogenesis from mutant ATXN1.

This anxiety-like phenotype, like motor and cognitive deficits, depends on the length of the polyQ tract: we did not observe increased anxiety in *Atxn1*^*78Q/2Q*^ mice, another SCA1 knock-in line that globally expresses mutant ATXN1 with fewer polyQ repeats.

Surprisingly, both lines of SCA1 knock-in mice consumed more sucrose than their wild-type littermate controls in the sucrose preference test—the opposite of what we would expect from a depressive-like phenotype. There are several possible explanations. First, SCA1 knock-in mice and some patients with SCA1 demonstrate weight loss and increased metabolic consumption^15,26^. Thus, increased sucrose preference may be a way for the SCA1 knock-in mice to meet their caloric needs. Alternatively, studies have also implicated the cerebellum, and in particular granule neurons, in the encoding of reward in mice^49^, suggesting that pathological changes caused by expression of mutant ATXN1 in granule neurons may alter the reward properties of sucrose. Patients with ataxia have demonstrated increased impulsivity and compulsivity which correlated well with depression^50^ indicating that cerebellum contributes to reward processing in humans as well^51,52^. The effect of mutant ATXN1 on the role of the cerebellum in regulating mood and encoding reward may account for the unexpected behavior of SCA1 knock-in mice in the sucrose preference test.

Knock-in mice are genetically similar to patients with SCA1 in that they have one normal and one expanded allele and have physiological levels and a physiological spatial and temporal pattern of mutant ATXN1 expression. However, symptoms in mice only occur at very long repeat lengths, whereas in human patients over 39 uninterrupted polyQ repeats causes disease. The longest repeat length reported in a human is 82Q, in a patient with juvenile SCA1 that is more severe than adult form of disease. While the longer polyQ requirement in mice is likely due to their shorter lifespan and other species differences, an alternative interpretation is that knock-in mouse models of SCA1 more accurately model juvenile form of SCA1. This would mean that any findings in these mice, including our results presented here, might have translational value mostly for the juvenile form of SCA1. In addition, the 154Q allele is longer than any found in humans, and the larger polyQ region could affect the ATXN1 protein function in additional, unpredictable ways.

In addition to its well-described role in motor control, the cerebellum also contributes to regulation of cognition and affect^53^. However, in contrast to SCA1 motor and cognitive deficits, the anxiety-like phenotype seen in *Atxn1*^*154Q/2Q*^ mice does not seem to be caused by cerebellar pathology. In fact, *ATXN1[82Q]* mice-SCA1 transgenic line that overexpresses mutant ATXN1 *only* in cerebellar Purkinje cells-exhibited attenuated anxiety. As levels of light can influence performance in EPM^33^, it is possible that increasing the light would prevent/moderate this phenotype. However, similarly attenuated anxiety is reported in Lurcher mice, another mouse model in which cerebellar Purkinje cells are affected^54^. In Lurcher mice, the attenuation of anxiety-like behavior was accompanied by an increase in stress-induced blood corticosterone levels, suggesting that attenuated anxiety may be actually caused by increased stress caused by testing^36^. However, this does not seem to be the case in *ATXN1[82Q]* mice as we have found that both basal and stress-induced blood corticosterone levels are decreased in *ATXN1[82Q]* mice, although this difference was not statistically significant. The attenuation of anxiety in *ATXN1[82Q]* mice further supports the finding that cerebellar damage can lead to disinhibition, reminiscent of the human cerebellar cognitive affective syndrome (CCAS), which leads to disinhibition, impulsiveness and risky behavior^23^.

Purkinje cell limited overexpression of mutant ATXN1 attenuated anxiety in transgenic *ATXN1[82Q]* mice, whereas global expression of mutant ATXN1 in knock-in mice increased anxiety. While interpreting these results, it is important to keep in mind differences in the levels and patterns of mutant ATXN1 expression. *Atxn1*^*154Q/2Q*^ mice express mutant ATXN1 at physiologically-relevant levels under the endogenous promotor, while transgenic *ATXN1[82Q]* mice overexpress mutant ATXN1 protein ~ 30–60 fold^25^, which likely accounts for the more severe Purkinje cell and cerebellar pathology in transgenic mice compared to knock-in *Atxn1*^*154Q/2Q*^ mice^55^. This severe Purkinje cell dysfunction may explain the attenuated anxiety in *ATXN1[82Q]* mice. On the other hand, the opposite finding of increased anxiety in *Atxn1*^*154Q/2Q*^ mice and altered sucrose preference in both *Atxn1*^*78Q/2Q*^ and *Atxn1*^*154Q/2Q*^ lines may suggest that dysfunction and/or pathology outside the cerebellar cortex contribute to mood alterations in SCA1 knock-in mice. ATXN1 is expressed throughout the brain, including hippocampus, cortex, cerebellum, and hypothalamus, and it is possible that pathology caused by polyQ expanded ATXN1 in any of these regions contributes to SCA1-like mood alterations^16,48,56^. Indeed, a recent study implicated hippocampal dysfunction in neurobehavioral deficits described in SCA1 knock-in mice^57^. Future studies using conditional SCA1 knock-in mice or viral-mediated approaches, in which polyQ ATXN1 can be selectively expressed or deleted in distinct brain regions, will be able to identify the anatomical substrates of mood alterations in SCA1.

PolyQ expansion causes both gain and loss of ATXN1 function^44,45^. ATXN1 gain-of-function is thought to underlie cerebellar neurodegenerative changes in SCA1^31^, while ATXN1 loss-of-function—in particular in complex with the transcriptional repressor Capicua (CIC)—causes a spectrum of neurobehavioral phenotypes in mice and humans^16^. Our forced swim test results support and build upon the concept that loss of ATXN1 function causes mood alterations in mice, with decreased immobility in the full knock-out mice. In contrast, the increased sucrose preference seen in global SCA1 knock-in SCA1 lines is likely due to a gain-of-function, as we did not detect it in the ATXN1 null mice.

The effect of ATXN1 loss-of-function on anxiety in the elevated plus maze was more difficult to interpret—a 50% decrease in ATXN1 expression resulted in a subtle anxiety-like phenotype in *Atxn1*^+*/−*^ mice, while full knock-out of ATXN1 slightly attenuated anxiety. Looking at the ranges covered by the 95% confidence intervals, our results can support two main interpretations: either there is little to no effect of ATXN1 loss of function on anxiety, or 50% and 100% loss of function cause opposite effects. The latter interpretation is consistent with a recent study by Lu et al., which suggests that disrupting the ATXN1-CIC complex in development can cause hyperactivity and decreased anxiety^16^. Together with that study, our results support the concept that complete loss of ATXN1 causes developmental abnormalities that result in the hyperactivity and attenuated anxiety in mice. There is evidence that this may also be the case in humans, as large deletions on chromosome 6p22 that span *ATXN1* gene have been reported in human patients that exhibit autism spectrum disorder (ASD), and attention deficit hyperactivity disorder (ADHD)^58,59^.

On the other hand, 50% loss of ATXN1 function may be well tolerated during development but cause neurobehavioral problems, including anxiety-like behavior, in adulthood. This is consistent with recent studies indicating loss of ATXN1 as a risk factor for Alzheimer’s disease and showing that a decrease in ATXN1 contributes to Alzheimer’s disease pathology^60,61^. Future studies using conditional and constitutive knock-outs of ATXN1 would be able to address these questions.

In conclusion, we have found alterations in mood in existing SCA1 mouse lines, suggesting that mutant ATXN1 pathology may contribute to anxiety and depression seen in patients with SCA1.

## Methods

### Mice

*Atxn1*^*−/−*^ mice^27^ on a C57/Bl6 background were a gift from the laboratory of Dr. Huda Zoghbi. *Atxn1*^*154Q/2Q*^, *Atxn1*^*78Q/2Q*^ and *ATXN1[82Q]* mice were a gift from the laboratory of Dr. Harry Orr. *Atxn1*^*154Q/2Q*^ mice are on C57/Bl6 background, while *Atxn1*^*78Q/2Q*^ and *ATXN1[82Q]* mice were originally on FVB background. To minimize the effects of genetic background, *Atxn1*^*78Q/2Q*^ and *ATXN1[82Q]* mice were backcrossed onto a C57/Bl6 background. Mice were housed in a temperature- and humidity-controlled room on a 12 h light/12 h dark cycle with access to food and water ad libitum. 8- to 10-week-old mice went through a battery of tests, with the same order of testing for all mice (in order: elevated plus maze, forced swim test, sucrose preference). Whenever possible we tried to ensure similar numbers of male and female mice (*Atxn1*^*154Q/2Q*^ (F:M = 5:8)*, Atxn1*^*78Q/2Q*^ (F:M = 8:12)*, ATXN1[82Q]* (F:M = 5:8)*, Atxn1−/− *(F:M = 5:6))*.*

All animal experiments were performed in ethical compliance with the National Institutes of Health’s Guide for the Care and Use of Laboratory Animals and were approved by the ethical University of Minnesota Institutional Animal Care and Use Committee (protocol number 1810-36435A).

### Corticosterone measurements

Corticosterone concentration was determined in total plasma from 7 pairs of *ATXN1[82Q]* mice and littermate WT controls (F:M = 5:2). Basal/minimally stressed corticosterone control measurements were obtained through tail clip bleed procedure in the morning (9–12 am) at least three days before stress induction. We collected samples in the morning due to lower corticosterone levels, and kept the time consistent between groups. After mice were transferred into the procedure room, we collected blood using tail snip within ~ 3 min using EDTA-coated capillary tubes that were then centrifuged for 25 min at 14.000 rpm at 4 °C. Plasma was diluted 1:100 using diluent supplied in Corticosterone Double Antibody RIA Kit (MP Biomedicals). Mice were allowed three days to recover from stress before stress-level blood samples were collected.

Stress level blood samples were collected after 15 min of restraint stress. We collected blood after decapitation. After centrifugation, plasma was diluted 1:200 in diluent. Corticosterone levels were determined by I^125^ radioimmunoassay using the *Corticosterone Double Antibody RIA Kit* in duplicate.

### Mood testing

#### Elevated plus maze

The maze was approximately 76 cm across and consisted of two open arms (6.3 cm × 34.2 cm) and two closed arms (6.3 cm × 34.2 cm with 19 cm tall opaque walls) extending from a 6.3 cm × 6.3 cm center area, raised 96 cm above the floor. The surrounding room was dark and the maze was lit by overhead lights. Light intensity in the open arms was 45–50 lx, and in the closed arms was 3–6 lx. Each mouse was placed in the center of the maze facing an open arm and allowed to explore for 5 min. During this time, the mice were tracked automatically using AnyMaze software and the time spent in each zone (open arms, closed arms, center area) was recorded. The maze was cleaned with 70% ethanol before each mouse^62^.

#### Forced swim test

Mice were placed in a transparent cylindrical container (diameter 18.4 cm, height 23.5 cm) filled to a depth of approximately 15 cm with water at 24–26 °C. They swam for 6 min before being removed from the water and placed in a cage containing absorbent material on a heating pad to dry off. Time immobile was tracked automatically using AnyMaze software (immobility was defined as a period of 85% or greater immobility lasting 250 ms or longer). The time spent immobile during the last 4 min of the test is analyzed^62^.

#### Sucrose preference test

Mice were singly housed in cages with cotton nestlets in order to allow for accurate measurement of each mouse’s water and sucrose consumption. Mice were acclimated to solitary housing for 3 days before the test. On the first day of testing, each mouse was given two bottles of water to test for any bias towards one side of the cage. On each day following this, each mouse was given one bottle containing water and another one containing either 2% or 4% sucrose. Bottles were weighed every 24 h to measure the amount of liquid consumed. Results for the 4% sucrose day, for which we saw the greatest differences between groups, are reported^62^.

All the results where we experienced technical difficulties, including poor video tracking during the EPM or FST, and leaking bottles during the sucrose preference test, were excluded from the final analysis.

### Statistical analysis

Wherever possible, sample sizes were calculated using power analyses based on the standard deviations from our previous studies, significance level of 5%, and power of 90%. Statistical tests were performed with GraphPad Prism. Data was analyzed using t-test or One-way ANOVA followed by post-hoc Tukey’s testing, and estimation statistics to present effect sizes and 95% confidence intervals.

## Data Availability

All data will be freely available from authors upon request.
